# Composite resin reinforced with silk nanoparticles from Bombyx mori cocoon for dental applications

**DOI:** 10.1590/0103-6440202304950

**Published:** 2023-05-15

**Authors:** Adriana da Silva Torres, João Vinícios Wirbitzki da Silveira, Moisés de Matos Torres, Cintia Tereza Pimenta de Araujo, Rodrigo Galo, Simone Gomes Dias de Oliveira

**Affiliations:** 1 Department of Dentistry, Faculty of Biological and Health Sciences, Federal University of Jequitinhonha and Mucuri Valleys; Diamantina-Minas Gerais, Brazil.; 2 Institute of Science and Technology, Federal University of Jequitinhonha and Mucuri Valleys; Diamantina- Minas Gerais , Brazil.; 3 Department of Dental Materials and Prothesis, Faculty of Dentistry of Ribeirão Preto, University of São Paulo, Brazil.

**Keywords:** Z350 resin, nanoparticle, dental restoration

## Abstract

The objective of this work was to evaluate the mechanical performance of Z350 resin composite modified with Bombyx mori cocoons silk nanoparticles for dental applications. Four experimental groups were analyzed G0% = Filtek Z350 resin composite (control); G1% = Filtek Z350 with 1% of silk nanoparticles; G3% = Filtek Z350 with 3% of silk nanoparticles; G5% = Filtek Z350 with 5% of silk nanoparticles. It was employed scanning electron microscopy, energy dispersive X-ray spectroscopy, X-ray diffraction, 3-point flexural strength test, Knoop hardness test, and surface roughness. From 3-point flexural strength tests the control group presented the best results G_0%_ = 113.33 MPa (±23.73). The higher flexural modulus was shown by groups G_3%_ = 29.150 GPa (±5.191) and G_5%_ = 34.101 GPa (±7.940), which are statistically similar. The Knoop microhardness test has shown statistical difference only among the G_3%_ group between the top 80.78 (± 3.00) and bottom 68.80 (±3.62) and no difference between the groups. The roughness test presented no statistical difference between the groups. The incorporation of silk nanoparticles reduced the flexural strength of Z350 resin composite. The surface roughness and microhardness tests showed no changes in any of the groups studied.

## Resumo

O objetivo deste trabalho foi avaliar o desempenho mecânico da resina composta Z350 modificada com nanopartículas de seda Bombyx mori cocoons para aplicações odontológicas. Quatro grupos experimentais foram analisados: G0%) Resina Z350 apenas (grupo controle); G1%) Reforço com 1% de nanopartículas de seda; G3%) Reforço com 3% de nanopartículas de seda; e G5%) Reforço com 5% de nanopartículas de seda. Foi empregado microscopia eletrônica de varredura, espectroscopia de energia dispersiva de raios X, difração de raios X, teste de resistência à flexão de 3 pontos, teste de dureza Knoop e rugosidade superficial. Nos testes de resistência à flexão de 3 pontos o grupo controle apresentou melhores resultados G0% = 0.113 GPa (±0.024). O maior módulo de flexão foi demonstrado pelos grupos G_3%_ = 29.151GPa (±5.191) e G_5%_ = 34.102 GPa (±7.94), que são estatisticamente semelhantes. O teste de microdureza Knoop mostrou diferença estatística apenas entre o grupo G3% entre os 80.78 superiores (± 3.00) e os 68.80 inferiores (±3.62). Não há diferença entre os grupos. O teste de rugosidade não apresentou diferença estatística entre os grupos. A incorporação de nanopartículas de seda reduziu a resistência à flexão da resina composta Z350. Os testes de rugosidade superficial e microdureza não apresentaram alterações em nenhum dos grupos estudados.

## Introduction

Resin composites are the direct restoration material widely employed in dental clinics. The many improvements of these materials, such as inorganic loadings optimization (surface modification, size, distribution, and particle morphology)^1^, have provided the expansion of their use and acceptable durability of the restorations^2^. Mechanical stability is one of the requirements for clinical success in long-term restorations. If the load exceeds the material capacity to support occlusal loads, it can lead to cracking a failure of this material^9^. Though the resin properties amelioration, systematic reviews show that fracture is one of the reasons for restorative failure in posterior teeth. This type of failure results in the renovation or replacement of restorations, which leads to higher wear and loss of dental structure^3^. A meta-analysis has shown that inside a ten-year observation period, at least 5% of resin composite posterior restorations can be one of the failure of dental restoration^10^. Many times the mechanical properties can be improved by increasing the amount of reinforcement in a resinous matrix^11^. Given the above, some studies suggest a resin modification aiming to deal with this failure^4^.

The silk from Bombyx mori silkworm is a protein polymer with high mechanical resistance, high biocompatibility, processing flexibility^5^, and resistance to chemicals and microorganisms^6^. This material has been in the health field as surgical sutures^7^, scaffolds for tissue regeneration, cell culture substrates, and drug delivery systems. Besides, silk is a very versatile material, and can be transformed in films, hydrogels, spheres, and frameworks^5^.

A high content of nanoparticles does not always lead to more desirable mechanical properties^12^. Because of this, it is mandatory to perform tests with different reinforcement concentrations, in order to evaluate, which, one brings the most adequate result. Then, the hypothesis of this study was that use of silk nanoparticles as a dispersed phase in dental resins can lead to improvements in flexural strength, hardness, and modulus of elasticity. Thus, the objective is to evaluate the mechanical performance of resin composite Z350 modified with Bombyx mori cocoons silk nanoparticles at different concentrations for dental applications.

## Materials and Methods

### Synthesis of silk nanoparticles from Bombyx mori cocoons^8^

The process of degumming, where the external layer of sericin is removed, was performed using 5g of torn silkworm cocoons. They were immersed for 30 min in a solution containing 5g of anhydrous sodium carbonate (Batch: 88682, Dinâmica, Química contemporânea Ltda., SP, Brazil) and 1L of deionized water at 100 °C. This procedure was repeated twice, and the degummed silk was rinsed with deionized water and oven dried at 40 ºC (SL-104/30, Solab, SP, Brazil) until constant weight.

The process of silk dissolution was performed by mixing 5g of dried degummed silk in a solution containing 1 mol of anhydrous calcium chloride (Batch: 212377, Synth, Labsynth, SP, Brazil), 2 mol of ethanol (Batch: 140548, Dinâmica, Química contemporânea Ltda., SP, Brazil) and 8 mol of deionized water. This mixture was heated up to 90 ºC using a thermal mantle (TE-0851, Tecnal, SP, Brazil) during 2 hours. It was used a reflux trap in order to avoid solvent loss due to evaporation. The final solution was centrifuged (Sorvall Legend Mach 1.6R, Thermo Scientific, MA, USA) for 20 min at 4000 rpm.

For the dialysis procedure, the solution was distributed in small flasks containing a semi-permeable cellulose acetate membrane (Unifil, Filtering Membrane, PR, Brazil). These flasks were submerged in deionized water during 96 hours, and the water changed each 24 hours. This process allows the ions removal and the retention of the nanoparticles.

The solution containing approximately 5% of silk (w/v) was slowly pipetted (a drop per second) in an aqueous solution of acetone (Batch: AC0224, Dinâmica, Química Contemporânea Ltda., SP, Brazil) 75% (v/v). The suspension was centrifuged for 1 hour at 4000 rpm, and the supernatant was discarded. It was added a small aliquot of water in the precipitate. In order to obtain a complete dispersion of the nanoparticles, the suspension was stirred in a Vortex stirrer (VX-28, Warmnest, SP, Brazil) during 20 seconds and submitted twice to a 55 W ultrasonic mixer (Q55 Sonicator, Qsonica, CT, USA) for 30 seconds and 40% amplitude. The suspension was then centrifuged for 1 hour at 4000 rpm. The processes of centrifugation and resuspension were repeated. Finally, the material was lyophilized to remove water.

### Characterization of silk nanoparticles

*Scanning electron microscopy (SEM) and energy-dispersive X-ray spectroscopy (EDS)*

The analysis of scanning electron microscopy and the energy dispersive X-ray spectroscopy were performed in an electron microscope (Tescan, Vega3 LMH, Czech Republic) operated at 30 kV. The dried sample was attached in a carbon double-faced tape supported in a metallic stub. The sample was sputtered with gold (Q150RS, Quorum Technologies Ltda., UK). The morphology was evaluated by SEM and the magnification, voltage, and resolution are described in each image. The composition was determined by EDS using the same equipment with an attached analyzer (Oxford Instruments, UK).

###  X-ray diffraction analysis (XRD) 

The samples were submitted to X-ray diffraction analysis in a diffractometer (Shimadzu, XRD-6000, Japan) using CuKα radiation with wavelength (λ) of 1,54056 Å (40 kV and 30 mA) and scanning speed of 2°/min, in the interval 2θ from 10° to 80°.

###  Preparation of the samples 

The samples were prepared with commercial resin composite (Batch: 1819400646, Filtek Z350 XT® 3M ESPE, St. Paul, MN, USA) and grouped in four groups according to the percentage of loaded nanoparticles in its composition: G_0%_ = commercial resin (control); G_1%_ = modified resin with 1% of silk nanoparticles; G_3%_ = modified resin with 3% of silk nanoparticles; G_5%_ = modified resin with 5% of silk nanoparticles.

The samples were manipulated in a dark chamber, using yellow light. The nanoparticles were weighted and added to the commercial resin. They were mixed until a complete homogenization over a glass plate using a spatula 70. After the mixture, the modified resins were inserted in different-sized molds, according to the requirementsfor the mechanical tests, pressed , and then polymerized. Standardized light curing was obtained using a radiometer (RD-7, Ecel, SP, Brazil) to measure the intensity of visible light emitted by the curing light (Optilight LD MAX, Gnatus, SP, Brazil) with an average irradiance of 600 mW/cm^2^.

### Mechanical properties characterization

###  3-Point flexural strength and flexural modulus 

The samples were grouped as shown previously (n = 10/group) and then inserted in a top-opened silicon matrix mold (2 x 2 x 25 mm). They were protected with a polyester cover and the set was pressed with a 500g load for 1 minute. The incident light source for the photopolymerization (Optilight LD MAX, Gnatus, SP, Brazil) was kept in five positions from the top of the rectangular sample. The samples were stored (Fanem, SP, Brazil) under heating at 37 ºC immersed in distilled water for 24 hours before testing.

The 3-point flexure strength evaluation was performed using standardized method (ABNT NBR ISSO 4049:2017 Dentistry - Polymer-based restorative materials), which regulates restorative dental materials tests. An universal testing machine (EZ - LX, Shimadzu, Kyoto, Japan) with a space between the base cylinders where the specimen was supported of 20mm, an indenter of 2mm in diameter and a speed of 1mm/min. The deflection curves were registered using a software. The flexure strength was calculated based on the load peak, length and cross-sectional area from the samples. The flexural modulus was determined from the initial linear slope from the load-displacement curves.

###  Knoop surface microhardness test 

The samples (n = 5/group) were produced from a Teflon ring-shaped mold of 4 mm diameter and 2 mm height. They were kept between two polyester stripes and pressed using a 500g load for 1 minute. In the sequence, they were photopolymerized (Optilight LD MAX, Gnatus, SP, Brazil) by 40 seconds from the top, and stored (Fanem, SP, Brazil) under heating at 37 ºC immersed in distilled water for 24 hours. The finishing and polishing procedures were executed with 600, 800, 1200, and 2000 silica carbide sandpaper disks (3M Espe, MN, USA). Each disk was employed for 1 min at 400 rpm in a metallographic polishing machine (Fortel, PLF, SP, Brazil) under water irrigation.

Knoop microhardness was measured using a microdurometer (HMV-2, Shimadzu, Kyoto, Japan) under a 50g load during 30 seconds. There were measured 5 hardness tests in each portion, superior and inferior, from the sample. In addition, each indentation had a 1 mm-distance between them. The average number from the readings was reported as sample hardness.

###  Surface roughness 

The samples (n = 10/group) were produced in a 5 mm diameter and 2 mm height silicon cast with a single opening. Over the superior portion, polyester stripes were placed, and a 500 g load was applied for 1 minute. They were photopolymerized (Optilight LD MAX, Gnatus, SP, Brazil) by 40 seconds from the top, and stored (Fanem, SP, Brazil) under heating at 37 ºC immersed in distilled water for 24 hours. The finishing and polishing procedures were executed with 600, 800, 1200, and 2000 silica carbide sandpaper disks (3M Espe, MN, USA). Each disk was employed for 1 min at 400 rpm in a metallographic polishing machine (Fortel, PLF, SP, Brazil) under water irrigation.

A surface roughness tester (Huatec SRT-6210, Instruments Co. Guangzhou Landtek Ltd, China) measured the average surface roughness (Ra) and the standard deviation (SD). There were obtained three parallel measurements from different places along the 0.8 mm length from the superior surfaces of the sample, the maximum measuring range being 300 µm (± 150 µm). The average surface roughness is so-called due to the fact that this device records the average between the peaks and troughs of the surface of the material to be studied.

### Statistical analysis

It was observed from Shapiro-Wilk normality test that the distribution was not normal for any of the evaluated mechanical tests. Then, it was employed Kruskal-Wallis nonparametric tests and the Mann-Whitney complimentary test in order to verify if there would be differences among the groups for the flexure strength, flexural modulus, and microhardness results. For the surface roughness test, it was used exclusively the Kruska-Wallis test. It was considered a significance level of 95%.

## Results

### Silk nanoparticles characterization

**Figure 1:**
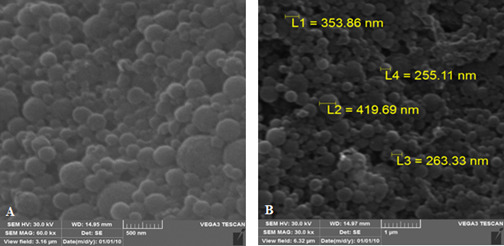
A and B SEM images from silk nanoparticles with spherical shape and 500 nm and 1000 nm scales, respectively.

According to the SEM analysis Figure 1 A and B, it was observed agglomerated nanoparticles with spherical conformation. They presented wide distribution on the diameters. All the registered measurements were below 1000 nm. In Figs. 1A and 1B a magnification of 60.000X and 30.000X was used, respectively.

**Figure 2:**
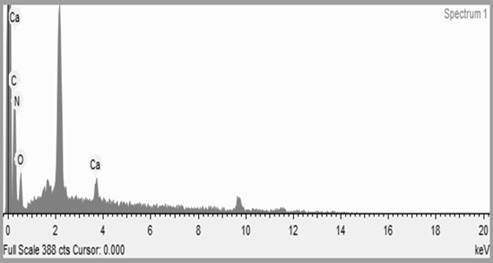
EDS spectra and characteristic peaks found in the silk nanoparticles structure.

According to the Figure 2, four main elements were identified. In molar percentages, carbon (26.7%), nitrogen (26.2%) and oxygen (26.2%) are the major components due to the chemical composition of fibroin. It was also found 1.3% of calcium, as residue from dissolution process.

**Figure 3:**
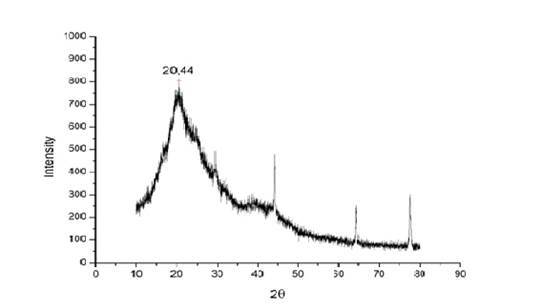
X-ray diffractogram from silk nanoparticles sample and a characteristic peak at 20º.

The Figure 3 shows the DRX diffractogram. An important peak found at 20° is related to the crystalline region from the silk polymeric structure. It is also important to observe the peaks at 44°, 64° and 78° that are related to the aluminum peaks from the metallic stub where the sample was placed, and there was not possible to eliminate its interference.

### Mechanical Properties

The results from 3-point flexural strength and flexural modulus are shown in Table 1 The addition of silk nanoparticles reduced the flexural strength of Filtek Z350. The flexural strength of G0% was significantly higher than other groups. G5% showed flexural strength significantly lower than other groups.

**Table 1:** Mean and standard deviation values of 3-point flexural strength and flexural modulus (MPa) for studied groups.

Group	Flexural strength (MPa)	Flexural Modulus (GPa)
G_0%_	0.113±0.020^a^	19.612±2.93^a^
G_1%_	0.088±0.001^b^	21.283±2.07^ab^
G_3%_	0.084±0.009^b^	29.150±5.19^bc^
G_5%_	0.076±0.007^c^	34.101±7.94^c^
p-value	0.001	0.005

Similar lowercase letters indicate statistical similarities between resin composites.

The addition of silk nanoparticles increased the flexural modulus of Filtek Z350. The G5% flexural modulus was significantly higher than the G0% and G1%. The G3% flexural modulus was significantly higher than G0%.

The results related to the microhardness test are presented in Table 2. There was no statistical difference observed among the G_0%_, G_1%_, G_3%_, and G_5%_ groups. As explicated by the table, the only statistical difference was between the top and the bottom of the G_3%_ group, where the microhardness from the top surface (80.78±3.00) was higher than the bottom surface (68.8±3.62).

**Table 2:** Mean and standard deviation values of Knoop surface microhardness for studied groups.

	G_0%_	G_1%_	G_3%_	G_5%_	p-value
Bottom	75.68±6,08^aA^	71.9±4.40^aA^	68.8±3.62^aA^	72.98±2.92^aA^	0,753
Top	80.24±1,96^aB^	83.54±4.20^aB^	80.78±3.00^bB^	75.52±1.37^aB^	
p-value	0.496	0.093	0.034	0.454	

Similar uppercase letters indicate statistical similarities between resin composites.

Similar lowercase letters indicate statistical similarities between top and bottom.

The acquired data have not presented significant differences among the groups, as observed in Table 3. The obtained p-values for average roughness (Ra) and root mean square (Rq) were higher than 0.05. Also, equal lowercase letters indicate that there is a similarity among the groups.

**Table 3:** Mean and standard deviation values of surface roughness for studied groups.

Group	Ra	Rq
G_0%_	1.19±0.54^a^	1.53±0.91^a^
G_1%_	1.29±0.44^a^	1.63±0.64^a^
G_3%_	1.30±0.57^a^	1.59±0.81^a^
G_5%_	1.69±0.91^a^	2.22±1.28^a^
p-value	0.527	0.371

Similar lowercase letters indicate statistical similarities.

## Discussion

A commercial resin reinforced with spherical crystalline silk nanoparticles, with different concentrations, was evaluated according to its mechanical properties. It is known that the silk fiber of the silkworm is a semi-crystalline polymer formed by crystalline and amorphous phases. The first presents two structures: silk I and silk II. The crystal structure is mainly composed of silk II as a result of the β sheet conformation^13^. After hydrolysis and purification, it is possible to obtain highly crystalline particles from Bombyx mori silkworm cocoons.

From EDS characterization of silk nanoparticles, it was observed the presence of four chemical elements. Considering the presence of carbon, nitrogen, and oxygen, expected from the fibroin polymeric structure, there was an unexpected presence of calcium. Calcium was found in small amounts probably due to remained chemicals from the synthesis process.

The principal peak found from DRX analysis occurs at 20° and is related to the β-sheet (silk II) crystalline phase of fibroin. It acts as a main structural constituent and is responsible for the superior mechanical properties of the silk^14^. Iridag and Kazanci^15^ observed this same behavior. According to the Braggs's Law, it is possible to determine the lamellar interplanar distance that results in a planar distance of 4.34 Å, approximately.

From the flexural strength test, it was observed a higher flexure resistance of unmodified resin (control group) than the modified ones. As more reinforcement was added to the resin, higher was the reduction in the flexural resistance, when compared to the control group. Only the nanoparticles incorporation of 1% and 3% have not presented any significant difference in resistance. Probably the absence of effect is due to the nanoparticle’s agglomeration^16^. It can lead to the formation of micrometric agglomerations and poorly dispersed nanoparticles in the matrix. Another factor that can contribute directly to the flexure strength is the bubble incorporation^17^ during the manual mixing of the nanoparticles.

Higher nanoparticle amounts in the G_3%_ and G_5%_ groups produced and incremented on the flexural modulus, which means low strain before breakage. The resin composites can undergo deformation in a permanent and progressive way, due to the application time of a load once these materials have a viscoelastic behavior^18^. The G_0%_ group was statistically similar to the G_1%_ group, having a lower flexure modulus. This similarity was not observed when comparing them with the two other groups.

The simultaneous effect of conversion and structure on mechanical properties must be considered^19^. The higher concentration of BisGMA when mixed with UDMA and TEGDMA reduced the degree of conversion of the composite, but the flexural strength and fracture toughness were maintained^20^. In this way, the reduction of the conversion and the crosslinking density can increase the number of rigid molecules of low mobility, promoting change in the values of flexural strength^19^.

The material hardness can be changed by the size, shape, fraction, and composition of the reinforcement^21^. Also, the hardness can be modified by the organic matrix structure^22^. However, the Knoop microhardness results have not shown differences among the evaluated groups. When microhardness from top and bottom surfaces are compared, only the G_3%_ group presented statistical differences. The reported top surface values (80.78±3.00) were higher than the bottom (68.8±3.62). There is a correlation between resin conversion degree and microhardness values^23^. An alteration in the polymerization degree can lead to a significant change in the hardness^24^. Probably it may have occurred with the G_3%_ group, causing the difference in the acquired results.

The surface roughness of the resin composite is influenced by the size, hardness, and concentration of the reinforcement material^25^. The incorporation of silk nanoparticles in the resin matrix did not show changes in surface roughness. Even with increasing fillers quantities. The silk nanoparticles incorporation in the resin matrix has not evidenced alterations in surface roughness. Even with the increased amounts of nanoparticles. This result is adequate, once the surface roughness increment could lead to more susceptibility to staining^26^, recurrent cavities, and plaque retention^27^.

A limitation of the study was the manual pipetting of the dialysis solution into the 75% aqueous acetone solution. This may have led to a wide-diameter size distribution of nanoparticles. A narrower size could have been produced if regular droplets could be produced. Another limitation is that Filtek Z350 is a high-filler resin composite and the addition of more fillers makes it even more difficult and increases the viscosity of the material as higher concentrations of nanoparticles were incorporated. In addition, an important limitation is related to the manual mixing of the nanoparticles with the resin, since the agglomeration of the nanoparticles is always a challenging problem to be avoided. Mechanical mixing and vacuum should be considered in future studies with silk nanoparticles, as well as the performance of other types of mechanical tests. In addition, the use of nanoparticles as drug delivery systems in dentistry is feasible.

It can be concluded that the control group G0% had better performance in the flexion test. The incorporation of silk nanoparticles showed a higher flexural modulus for groups G3% and G5%, which is not a desired result, since a lower elastic modulus reduces the chances of failure, as it absorbs more masticatory forces. There were no changes in surface roughness and microhardness for any of the groups studied.
